# Therapeutic Efficacy of a Very Low/Low Dose of Lenvatinib in Advanced Radioiodine-Refractory Thyroid Cancer: A Real-World Series from a Single Center

**DOI:** 10.3390/cancers17142372

**Published:** 2025-07-17

**Authors:** Vittorio Oteri, Fiorenza Gianì, Giulia Sapuppo, Stefania Panebianco, Ilenia Marturano, Giusi Blanco, Pasqualino Malandrino, Marco Russo, Francesco Frasca, Gabriella Pellegriti

**Affiliations:** 1 Endocrinology Section, Department of Clinical and Experimental Medicine, University of Catania, Garibaldi-Nesima Hospital, 95122 Catania, Italy; research@droteri.it (V.O.); dottoressa.panebianco@gmail.com (S.P.); f.frasca@unict.it (F.F.); gabriella.pellegriti@unict.it (G.P.); 2 Department of Medicine and Surgery, “Kore” University of Enna, 94100 Enna, Italy; fiorenza.giani@unikore.it; 3 Endocrine Unit, Garibaldi-Nesima Hospital, 95122 Catania, Italy; imarturano@arnasgaribaldi.it (I.M.); marcorusso@arnasgaribaldi.it (M.R.); 4 Oncology Unit, Garibaldi-Nesima Hospital, 95122 Catania, Italy; gblanco@arnasgaribaldi.it; 5 Medical Oncology, Department of Clinical and Experimental Medicine, University of Catania, 95123 Catania, Italy

**Keywords:** lenvatinib, tyrosine kinase inhibitor (TKI), advanced thyroid cancer, radioiodine-refractory thyroid cancer, low dose, case series, outcome

## Abstract

**Background:** Lenvatinib is a receptor tyrosine kinase inhibitor indicated for advanced radioiodine-refractory thyroid cancer (RAI-RTC). It is recommended to start at 24 mg per day; however, in patients who are at risk of severe adverse events, it may be reasonable to start at lower doses. **Patients and Methods:** We included 15 patients with RAI-RTC who started lenvatinib at a very low/low dose and evaluated the efficacy and safety. **Results:** Eight patients (53.3%) did not show progression of the disease, and about half of the patients (53.3%) were alive at the last follow-up visit. Up to 26.6% of patients achieved a partial response to therapy, with a notable volume reduction in the local and metastatic lesions. However, 80% of patients experienced adverse events, mainly of a moderate grade. **Conclusions:** Although these findings are based on a small sample size and a single-center study, treatment with lenvatinib at very low/low doses in fragile patients seems to be a promising strategy for the management of RAI-RTC, balancing effective disease control with a favorable safety profile.

## Simple Summary

Managing the treatment of advanced RAI-RTC (advanced radioiodine-refractory differentiated thyroid cancer) with progressive disease remains a clinical challenge in the presence of comorbidities or locally advanced (with invasion of the trachea, the esophagus, and/or the carotid artery) and unresectable or metastatic progressive tumors. Initiating lenvatinib at a low or very low intensity in these patients seems to be a promising approach, balancing effective disease control with a favorable safety profile. The aim of our paper is to describe the therapeutic efficacy and safety of a very low (4 mg/day) or low (4–10/day) lenvatinib dose in 15 patients with RAI-RTC. In our experience, lenvatinib, even at very low doses, can achieve significant tumor reduction and extend progression-free survival, all while minimizing severe adverse effects.

## 1. Introduction

Thyroid cancer affects approximately 10.1 women and 3.1 men per 100,000 persons worldwide. Among the histologic types, differentiated thyroid cancer (DTC), which primarily includes papillary thyroid carcinoma (PTC) and follicular thyroid carcinoma (FTC), constitutes around 95% of all thyroid carcinomas [1,2]. Despite a 90% disease-specific survival rate, 10–15% develop recurrence/metastases, with 60–70% acquiring radiorefractoriness [3], either initially or during follow-up [4]. The impact of radiorefractoriness on the prognosis is negative, with a median life expectancy from diagnosis of only 3–5 years, and a median 10-year survival rate of about 40% [5].

Lenvatinib is a multi-kinase inhibitor that has demonstrated notable efficacy in extending the median progression-free survival (PFS) for patients diagnosed with advanced radioiodine-refractory thyroid cancer (RAI-RTC). A registrational study (phase III SELECT trial) showed that lenvatinib yields a median PFS of 18.3 months (95% CI, 15.1 to not estimable), significantly longer than the 3.6 months (95% CI, 2.2 to 3.7) observed with placebo [5,6].

A phase II study established the initial dosage of lenvatinib at 24 mg/day [7,8]. Despite this determination, real-world evidence has shown that a considerable proportion of patients necessitated dose reductions or interruptions ranging up to 86% [6,9,10,11] to effectively manage adverse effects. The most commonly reported adverse events were diarrhea, hypertension, fatigue, and proteinuria [6]. Additionally, rapid tumor shrinkage may trigger rare but severe fistula formation and tumor-related bleeding [12].

Managing the treatment of advanced RAI-RTC with progressive disease remains a clinical challenge in the presence of comorbidities or locally advanced and unresectable tumors (involving the trachea, esophagus, and major neck vessels) in terms of the risk of complications due to the anti-VEGF activity of the drug, such as upper airway fistula formation, which can be life-threatening [13].

With this in mind, starting lenvatinib at a lower dose may be preferable, especially for patients with compromised performance status, advanced age, or comorbidities that lenvatinib therapy could worsen.

Lenvatinib is typically not subject to therapeutic drug monitoring (TDM) in clinical practice, despite its potential utility in optimizing the dosage for patients. This differs from the management of drugs with a narrow therapeutic index, for which TDM is essential to balance efficacy and avoid toxicity. While a priori TDM could theoretically help in tailoring lenvatinib dosing based on pharmacokinetic (PK) and pharmacodynamic (PD) models, the variability in terms of patient responses, lack of established PK/PD markers for clinical use, and logistical challenges prevent its widespread application in lenvatinib therapy [14].

For this reason, lower-dosage regimens should be evaluated for efficacy and safety; however, to date, only two published real-world studies can be found in the literature on this specific topic. Although this research uses a small sample size and a single-center study, the aims were to report on the therapeutic efficacy and safety of a very low (4 mg/day) or low (>4–10 mg/day) lenvatinib dose, relative to the label recommendation of 24 mg/day, in 15 advanced RAI-RTC patients.

## 2. Patients and Methods

We conducted a retrospective cohort study of 15 adult patients with a diagnosis of advanced RAI-RTC who were treated with a very low/low starting dose of lenvatinib at our institution (Endocrine Unit of Garibaldi-Nesima Hospital, Catania, Referral Center for Thyroid Cancer) between November 2020 and May 2025, with a median follow-up time of 7.1 years from DTC diagnosis (IQR 3.3–12.1, min 0.8, max 24.0) [15,16].

Radioiodine (RAI)-refractory disease was defined by the following criteria: (1) metastatic disease that does not uptake iodine “ab initio” or entail loss of the ability to uptake RAI over time; (2) iodine uptake present only in some locations and absent in others; (3) reaching the maximum cumulative recommended dose of RAI (600 mCi/22.2 gigabecquerel (GBq)) or exceeding it cumulatively without signs of remission [17]; and (4) having locally advanced and/or metastatic progressive DTC.

The median age at diagnosis was 67.4 yrs (63.1–71.6). Most of the patients were diagnosed at stage IVA or IVB, with all of them showing locoregional or distant metastasis at the time of lenvatinib initiation. Eighty percent of the patients had previously been treated with radioiodine.

Almost all the patients (13/15, 86.7%) started lenvatinib for RECIST progressive disease, and the remaining 2/15 (13.3%) for bulky disease.

The starting dose of lenvatinib was individualized based on clinical considerations. In particular, very low or low doses (4–10 mg) were chosen for patients with locally invasive disease involving critical structures (e.g., trachea, esophagus) due to the increased risk of fistula formation or bleeding from rapid tumor necrosis. Other factors influencing the dose reduction included critical bone injuries, esophagitis, carcinomatous lymphangitis of the lung, brain and critical bone metastases, advanced age (median 74.6 years, IQR 69.7–78.9), and significant comorbidities such as rheumatoid arthritis. This cautious dosing approach aimed to minimize severe adverse events.

In all the patients, the starting dose was decided after a multidisciplinary discussion by a thyroid cancer tumor board and according to the literature data and the risk of local adverse events [18]. Almost all the patients (13/15, 86.7%) started lenvatinib at 4 mg/day, with one patient starting at 8 mg and another at 10 mg. All the patients were informed about the rationale for starting the therapy at a low dose compared to the 24 mg/day dose [6,10].

Clinical and biochemical follow-up was performed at baseline, 1 month, and then quarterly, except in specific cases.

The assessments included serum thyroglobulin (Tg) level and anti-Tg antibody measurements (AAT), neck ultrasound, and additional diagnostic imaging such as computerized tomography (CT) and magnetic resonance imaging (MRI) scans. In addition, locoregional therapies (e.g., radiotherapy, percutaneous ablation, surgical resection) were performed when required (e.g., for a single evolutive lesion). The treatment efficacy was assessed based on radiological reports according to the Response Evaluation Criteria In Solid Tumors (RECIST) guidelines, version 1.1 [19]. Treatment AEs were recorded and graded according to the Common Terminology Criteria for Adverse Events (CTCAE), version 5.0.

Ethical approval was obtained from the ethics committee of our medical center. Patient consent for publication was obtained by the authors via informed consent.

### Statistical Analysis

Categorical variables were expressed as frequencies and percentages. Normally distributed quantitative variables were expressed as means ± standard deviations (SDs), whereas non-normally distributed variables were expressed as medians and interquartile ranges (IQRs). The normality of the quantitative variables was assessed with the Kolmogorov–Smirnov test. The probability of developing an adverse event was assessed with the Charlson comorbidity index.

A *p* value < 0.05 was considered statistically significant for all the analyses. Data analysis was conducted using SPSS statistic software version 13.0 for Windows.

## 3. Results

### 3.1. Baseline Patient Characteristics

The clinical baseline and histopathological characteristics are summarized in Table 1, and individual patient details are shown in Table 2.

The median age at diagnosis was 67.4 years (IQR 63.1–71.6); ten of the patients were female (66.6%). Histologically, nine cases (60.0%) were PTC, five were FTC (33.3%), and one was a poorly differentiated carcinoma (PDTC) (6.7%). RAI treatment was performed for twelve patients (80%) who had previously undergone surgery, and the median cumulative dose was 250 mCi (IQR 175–440). Radioiodine refractoriness was reached after a median of 4.3 years (IQR 1.5–10.6). The median age at the start of therapy was 74.6 years (IQR 69.7–78.9).

Nine patients were tested for the presence of gene alterations. Two patients harbored a mutation in the NRAS gene, one patient harbored a mutation in the P53 gene, and another patient tested positive for BRAF V600E. One of the patients harbored concurrent RAS and Met alterations. Another patient had mutations in both the TERT and CHECK2 genes. No mutation in the gene panel was detected for three cases.

All the patients had metastatic lung disease, followed by bone disease (53.3%). Other sites, such as the brain and skin, were affected in two patients (13.4%) and one patient (6.7%), respectively. In addition, locoregional invasion was observed in nine patients (60%).

Three patients with unresectable local cancer started lenvatinib as neoadjuvant therapy. None of the patients had previously received tyrosine kinase inhibitor therapy (TKI), chemotherapy, and/or local external beam radiotherapy (EBRT). At the start of the lenvatinib treatment, the mean age was 74.6 years (range 69.7–79.0).

Regarding comorbidities, almost all the patients (12/15, 80%) were affected by hypertension (HTN); 8/15 (53.3%) patients also had dyslipidemia, 5/15 (33.3%) had diabetes mellitus (DM), 2/15 (13.4%) had previous myocardial infarction, 1/15 (6.7%) had chronic obstructive pulmonary disease, 1/15 (6.7%) had atrial fibrillation, 1/15 (6.7%) had heart failure, and 1/15 (6.7%) had a previous other cancer.

### 3.2. Treatments and Outcomes

**Figure 1:**
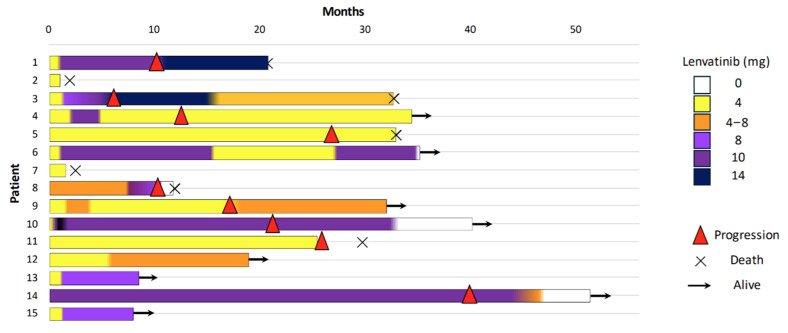
Swimmer plot showing the individual patient treatment timelines (n = 15). Each horizontal bar represents a single patient, with the color changes within the bar indicating modifications in the lenvatinib dose over time. (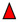) denotes disease progression, (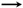) indicates patients still alive at last follow-up, and (×) represents death.

Details of the lenvatinib therapy and its effectiveness outcomes are shown in Table 3. We have included a swimmer plot displaying how much of a dose each patient was treated with (Figure 1).

The median duration of treatment was 25.1 months (IQR 5.2–25.1, range min–max 1.1–50.7).

**Figure 2:**
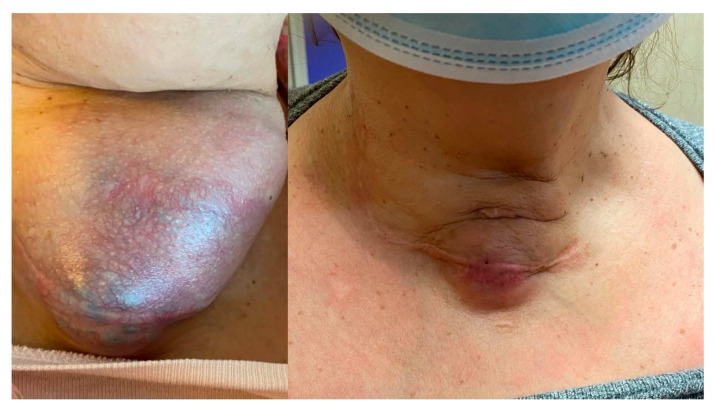
A notable reduction in the neck mass and skin tension in a patient (Case 8) after two months of lenvatinib therapy at 4 mg. Minimal fistula formation with emission of serous secretion occurred and lasted a few days. The Tg value was 19.54 ng/mL at baseline and 5.37 after two months.

Despite the low starting dose, 53.3% of patients did not show progression of the disease, and 8 out of 15 patients (53.3%) were still alive at the last follow-up visit. Additionally, 26.6% of the patients achieved a partial response, with reductions in the size of both local and metastatic lesions, according to the RECIST criteria (Table 2 and Figure 2).

The median time to therapy dose titration was 1.4 months (IQR 0.6–2.0) from the start of lenvatinib, and the maximum administered dose ranged from 8 to 14 mg (median maximum dose 6 mg, IQR 4–9). At the last follow-up visit, of the eight alive patients, three were still being treated with 4 mg alternating with 8 mg, and five patients had been escalated to higher doses (10 and 14 mg).

Progression-free survival (PFS) was evaluated using the Kaplan–Meier method. The estimated PFS rates at 6, 12, and 24 months were 86.7%, 78.8%, and 68.9%, respectively. The median PFS was 29.3 months.

### 3.3. Adverse Events

Overall, 12 patients (80.0%) experienced at least one AE of any grade. The most common AEs were hypertension and diarrhea, affecting five (33.3%) and three patients (20%), respectively. In our study, the dose reduction rate was 53.3% vs. the SELECT trial rate of 68–86%, which was probably due to the greater tolerability of the low dose. Other notable AEs included QTc prolongation, transaminase elevation, hemoptysis, fistula formation (without life-threatening sequelae), mucositis, hand–foot syndrome, proteinuria, and epistaxis, each occurring in a single patient. Most AEs were moderate in severity (grade 2) according to the CTCAE criteria (https://ctep.cancer.gov/protocoldevelopment/electronic_applications/docs/ctcae_v5_quick_reference_5x7.pdf, accessed on 30 January 2025). However, four patients (33.3%) experienced severe grade 3 AEs, including respiratory insufficiency, pleural effusion, and proteinuria. Three of these cases required hospitalization. Despite the presence of AEs, most patients tolerated the low-dose lenvatinib regimen, with only a few requiring dose adjustments or discontinuation.

Furthermore, we evaluated the probability of developing an adverse event according to the Charlson comorbidity index. Each one-point increase in this index is associated with a four-fold higher probability of developing an adverse event (OR = 4.20). However, this result is not statistically significant (*p* = 0.19), likely due to the small sample size (n = 15).

## 4. Discussion

Our study demonstrates that initiating lenvatinib treatment at a very low or low dose shows promising therapeutic efficacy and safety in patients with advanced RAI-RTC. Even though treatment was started at a very low intensity and the maximum dose reached was 14 mg (median maximum dose: 6 mg, IQR 4–9), 53.3% of the patients exhibited no disease progression, and 8 out of 15 patients (53.3%) were still alive at the last follow-up visit. The median duration of treatment of 25.1 months (IQR 5.2–25.1, range min–max 1.1–50.7) in our study further supports the long-term viability and sustainability of this dosing strategy, indicating that this therapeutic approach could maintain disease control and manage adverse events over a prolonged time.

For some patients, low-dose lenvatinib represented a rescue therapy, despite the risk of life-threatening sequelae. All the patients in our series were warned of possible side effects, including local life-threatening ones.

Remarkably, 26.6% of the patients achieved a partial response to therapy, resulting in a reduction in both the local and metastatic lesion volumes, even if the median maximum dose of lenvatinib reached for these patients was 4 or 8 mg daily. The median PFS was 29.3 months, longer than the 18.3 months in the registrational study.

In terms of the efficacy and tolerability, our findings are consistent with those of Jiang et al.’s [20] and Yamazaki et al.’s [21] studies. The former studied a cohort of 65 patients treated with lenvatinib at an initial and maintenance median dose of 10 mg/day; this regimen showed less dose reduction/discontinuation compared to the standard dose while maintaining a worthy PFS and OS. Following these results, Yamazaki et al. also demonstrated the better tolerability of lenvatinib started at a dose lower than 24 mg, with only a 10% difference in therapeutic efficacy [21]. They compared the efficacy and tolerability of a low dose versus 24 mg of lenvatinib in a case series of thirty-six patients with a response rate of 33% in the low dose group. For some patients, low-dose lenvatinib represented a rescue therapy, despite the risk of life-threatening sequelae. All the patients were warned of possible side effects, including local life-threatening ones.

Our data are not aligned in terms of safety with the findings of Yamazaki et al.: only three (20%) of our patients interrupted lenvatinib treatment vs. 67% of the low-dose group in Yamazaki et al.’s cohort. This discrepancy may be attributed to the different drug doses: in our study, patients started treatment at a dose of 4–8 mg/day, escalating up to a maximum of 10 (only one 14 mg/day for a short period); in their study, patients in the LD group started therapy at doses of 20, 14, or 10 mg/day. Similarly, Murakami et al. [22] conducted a retrospective analysis of 42 patients to evaluate the impact of prolonged lenvatinib treatment, aiming for the longest feasible duration with minimal discontinuation periods. Their findings, in line with our report, demonstrated that patients who maintained an average dose ranging from 4.56 to 7.97 mg experienced the most favorable outcomes in terms of safety and efficacy; indeed, this approach minimized the adverse events, enabling patients to continue therapy for extended periods.

Recently, Worden et al. [10] retrospectively analyzed a series of 308 RAI-RTC patients treated with first-line lenvatinib. Overall, 62% of patients started lenvatinib at the recommended starting dose (24 mg), and the remainder at 14 to 20 mg daily. At the last follow-up visit, 32% of patients permanently discontinued the treatment, mainly because of disease progression or death. Unfortunately, data on the effectiveness of the different dosages (24 mg vs. 14–20 mg/daily) were not provided.

A real-world series by Masaki et al. [23] evaluated the efficacy and limitations of lenvatinib therapy in 42 RAI-RTC patients, starting with a dose of 24 mg daily and a mean latest daily dose of 10 mg after a median follow-up of 15 months due to a high percentage of reductions/discontinuations of therapy. AEs were seen in all the patients, mainly hypertension, proteinuria, appetite loss, and diarrhea. In this study, some of the patients continued treatment thanks to a dose escalation or arranged interruptions, avoiding long interruptions due to severe AEs.

In this scenario, a post hoc analysis of the SELECT study [24] by Tahara et al. showed the negative impact of dose interruption on the efficacy of lenvatinib: the hazard ratios for PFS in the shorter (<10% of total treatment duration) and longer (≥10% of total treatment duration) dose-interruption groups were, respectively, 0.14 (95% CI 0.09–0.20) and 0.31 (95% CI 0.22–0.43).

Similar experiences have also been described for other tumors. Recent investigations have demonstrated that dose escalation can contribute to disease control if AEs are tolerable [25,26].

Our findings are consistent with a case series by Tang et al. [27], which described three patients with advanced hepatocellular carcinoma (aHCC) who experienced durable responses to very low doses of TKIs. Despite receiving a fraction of the FDA-recommended target doses, an eighth for sorafenib, a quarter for regorafenib, and a sixth for cabozantinib, the patients maintained prolonged disease control. Notably, their time to radiologic progression and overall survival exceeded the averages reported in the corresponding phase III clinical trials for these agents, underscoring the potential efficacy of low-dose therapy. This case series questions the practice of discontinuing subtherapeutic doses without first evaluating the clinical response, especially considering the non-cytotoxic mechanisms of TKIs and the uncertainty about their minimal effective doses. Applying this broader dosing paradigm to lenvatinib in TC, our study suggests that initiating treatment at low doses can also result in disease control and manageable adverse events. In both our study and that by Tang et al., patients benefited from lower TKI doses, which not only minimized the side effects but also allowed them to be treated for longer periods. Significantly, all three patients in their report not only achieved prolonged tumor control but also reported significant improvements in quality of life when maintained on lower, more tolerable doses.

Our results, given that 80.0% of patients experienced treatment-related adverse events, confirm that starting at a lower dose reduces the overall burden of these events and improves the quality of life for patients: notably, we had to interrupt the treatment with lenvatinib for only three patients to manage their AEs. In our series, hypertensive crises were observed in only 33% of cases, even if all the patients were treated for hypertension before starting lenvatinib therapy, which was probably due to close monitoring.

These findings strongly indicate that maintaining a lower, more manageable dose may contribute to a better quality of life for patients, allowing them to continue treatment with fewer complications. It would be interesting in the future to understand how the pharmacodynamics could be influenced according to age and weight.

In the context of TDM, lenvatinib represents an interesting case. While TDM is a well-established practice for medications with narrow therapeutic windows, such as antiepileptics or immunosuppressants, it is not commonly applied for this drug. Several factors contribute to this issue: the absence of a well-defined therapeutic range, the complexity of the drug’s multi-kinase inhibition mechanism, and the challenges in correlating blood concentrations with clinical outcomes [14]. In studying TDM for TKIs like lenvatinib, ultra-performance liquid chromatography/tandem mass spectrometry (UPLC-MS/MS) has become the reference method due to its high selectivity and efficiency [28] and because it is capable of high-throughput analysis in clinical settings. However, limitations such as the complex mobile phase composition, long analysis time, and complicated sample preparation exist. Other methods, such as capillary electrophoresis–UV and high-performance liquid chromatography–UV (HPLC-UV) [28,29], offer alternatives. Data show significant inter-individual variability in the trough concentrations of lenvatinib, and its relationship with the efficacy and adverse effects is still under investigation [28]. PK data from the SELECT trial provide some results, suggesting that in patients with lower body weight, the area under the curve (AUC) of lenvatinib is higher; this could explain the higher percentage of side effects in subgroups of patients [30].

In selected patients with comorbidities and/or advanced disease, TDM would offer additional benefit for dose management of lenvatinib in the future.

Moreover, unlike certain other targeted therapies where TDM can guide dose adjustments, lenvatinib dosing adjustments are primarily driven by the clinical response and the management of adverse events rather than the serum levels. Our study is particularly relevant because we started lenvatinib at a lower dose of 4–10 mg/day without relying on pharmacokinetic monitoring. Despite this, we observed significant tumor reduction and prolonged progression-free survival in a substantial proportion of patients. This suggests that even without TDM, careful clinical management, starting with lower doses in vulnerable populations, can achieve effective outcomes while minimizing adverse effects. This aligns with the broader understanding of the lenvatinib non-cytotoxic mechanism of action and raises the question of whether routine TDM would offer added benefits for this drug in the future.

This study has some limitations, as it is a single-center, nonrandomized, retrospective study including a small number of patients, but considering that RAI-RTC is a rare disease, these could be interesting data to be confirmed by other groups. Future research should focus on larger, prospective, randomized controlled trials, stratified by comorbidities and including a TDM arm, to confirm the efficacy and safety of low-intensity lenvatinib in patients with advanced RAI-RTC and to identify specific patient subgroups that may benefit most from this approach

It would also be valuable to investigate the potential for redifferentiation of thyroid cancer cells after treatment with lenvatinib, which could restore radioiodine uptake and allow for subsequent radioiodine therapy, as suggested by emerging studies [31,32].

## 5. Conclusions

Initiating lenvatinib at very low or low doses in fragile patients with unresectable locally advanced (involving invasion of the trachea, the esophagus, and/or the carotid artery) and/or metastatic progressive differentiated advanced RAI-RTC seems to be a promising strategy, balancing effective disease control with a favorable safety profile. This approach demonstrates that, even at very low doses, lenvatinib can achieve significant tumor reduction and extend progression-free survival, all while minimizing severe adverse effects. This study underscores the potential for personalized dosing regimens in enhancing patients’ quality of life and treatment outcomes.

This case series, despite its limitations (small sample size and single-center design), provides preliminary evidence that low-dose TKI therapy may still be effective. Accordingly, administering TKIs at lower doses may serve as a feasible therapeutic strategy in some patients with advanced thyroid cancer, particularly when no other treatment options are available.

## Figures and Tables

**Table 1:** Clinical and histopathological characteristics of the 15 RAI-RTC patients treated with a low dose of lenvatinib.

	n.	%
Patients (n.)	15	
Follow-up median (IQR) (years)	7.1 (3.3–12.1)	
Age at diagnosis median (IQR) (years)	67.4 (63.1–71.6)	
Female patients	10	66.6
Underwent surgery	13	86.6
Histotype		
Papillary	9	60
Follicular	5	33.3
Poorly differentiated	1	6.7
T status		
T1a	1	6.7
T2	5	33.3
T3a	5	33.3
T3b	1	6.7
Tx	3	20
N status		
N0	3	20
N1a	3	20
N1b	2	13.4
Nx	7	46.6
M status (at diagnosis)		
M1	11	73.3
M0	1	6.7
Mx	3	20
Stage		
I	2	13.4
II	2	13.4
IVB	11	77.3
ECOG performance status		
0	9	60
1	6	40

**Table 2:** RAI-RTC patient-specific characteristics.

Case	Ineligible for Full Dose	Started Dose	Maximum Dose Reached	Distant Metastasis and/or Local Invasion at Lenvatinib Start	Mutation (NGS Panel)	Status
1 (M)	Local invasion	4 mg	14 mg	Lung; local invasion; bones	N/A	Dead
2 (F)	Critical bone injury and esophagitis	4 mg	4 mg	Lung; skin; bones	N-RAS (PQ61R 38%)	Dead
3 (M)	Local invasion	4 mg	14 mg	Lung; local invasion	P53 (SER183TER 40%)	Dead
4 (M)	Local invasion	4 mg	10 mg	Lung; local invasion; bones	N/A	PR
5 (F)	Local invasion	4 mg	4 mg	Lung; local invasion; bones	N/A	Dead
6 (F)	Critical lung injury	4 mg	10 mg	Lung	No mutation found	PD
7 (M)	Local invasion	4 mg	4 mg	Lung; local invasion	N/A	Dead
8 (F)	Local invasion	8 mg	8 mg	Lung; local invasion	N-RAS (p.Gly12Val)	Dead
9 (F)	Carcinomatous lymphangitis of the lung	4 mg	8 mg	Lung; bones	N-RAS (Q61K 51%); PRR20A-MET fusion	PR
10 (F)	Age and comorbidities	4 mg	10 mg	Lung; bones	N/A	SD
11 (F)	Local invasion	4 mg	4 mg	Lung; local invasion	BRAF (V600 E)	Dead
12 (F)	Brain and critical bone metastasis	4 mg	8 mg	Lung; brain; bones	No mutation found	SD
13 (M)	Comorbidities (rheumatoid arthritis in therapy)	4 mg	4 mg	Lung	No mutation found	PR
14 (F)	Local invasion	10 mg	10 mg	Lung; local invasion	N/A	PD
15 (M)	Local invasion and brain metastasis	4 mg	4 mg	Lung; local invasion; bones; brain	TERT; CHECK2	SD

PR—partial response; SD—stable disease; PD—progressive disease.

**Table 3:** Treatments and outcomes of the 15 RAI-RTC patients treated with a low dose of lenvatinib.

	n.	%
Patients treated with radioiodine	12	80
Cumulative radioiodine dose, median (IQR)	250 (200–350)	
Radioactive iodine-refractory from diagnosis, years (median, IQR)	4.3 (1.5–10.6)	
Time to therapy augmentation, months (median, IQR)	1.4 (0.6–2.0)	
Dose reached, mg (median, IQR)	6 (4–9)	
Best response to therapy, months (median, IQR)	4.2 (3.4–9.0)	
Tg difference before and after lenvatinib at the last follow-up visit, percentage (median, IQR)	−59.9% (−94.9%–+5.5%)	
Alive at last visit	8	53.3
Progression	2	13.3
Stable	6	40

## Data Availability

Data are available in the Garibaldi Medical Center archive.
